# Unusual lesion in the splenium of the corpus callosum and coronavirus infectious disease-19

**DOI:** 10.1259/bjrcr.20200068

**Published:** 2020-07-03

**Authors:** Nivedita Agarwal, Rosella Martini, Giovanni Pedrotti, Sabino Walter Della Sala

**Affiliations:** 1 Section of Radiology, Santa Maria del Carmine Hospital, Rovereto (TN), Italy; 2 Center for Mind/Brain Sciences, University of Trento, Rovereto (TN), Italy; 3 Section of Anesthesia, Santa Maria del Carmine Hospital, Rovereto (TN), Italy

## Abstract

Coronavirus infectious diseases (COVID-19) is an emerging infectious disease that has taken a strong grip on the entire global community. The diagnosis *per se* is straightforward; however, the disease seems to affect multiple organs. Cytokine-storm, increased thromboembolic state, deranged overall homeostasis and aggressive medical treatment can cause a variety of lesions in the central nervous system. Interpretation of brain imaging findings will require a thorough understanding of the clinical status of the patient and treatment being offered, keeping in mind the several ways in which the viral pathogen, severe acute respiratory syndrome coronavirus-2 may interact with brain tissue. We present differential diagnosis of a MRI identified solitary lesion in the splenium of the corpus callosum, in a COVID-19 positive patient with altered mental status.

## Introduction

On March 11, 2020, the World health organization declared a pandemic outbreak caused by a novel coronavirus, severe acute respiratory syndrome coronavirus (SARS-CoV-2).^1^ Viral agents, a host of toxic substances including drugs and disturbed overall homeostasis may result in a variety of brain lesions that require neuroradiologic expertise for appropriate differential diagnosis in the coronavirus infectious disease (COVID-19) positive patients. A high propensity for thromboembolic events and a massive release of cytokines are recognized as severe complications of the disease that can lead to deadly complications. Aggressive medical treatment and mechanical ventilation is thereby proposed until dedicated therapies are developed. Patients are treated with multiple drugs such as sedatives, antivirals, antibiotics, anti epileptics and extended prophylactic treatment with anticoagulants is recommended at early stages of the disease.^2^ Such aggressive treatment may result in several brain parenchymal lesions which may or may not be directly related to SARS-CoV-2. We describe here the CT and MRI findings from a patient with COVID-19 where a lesion in the splenium with subsequent hemorrhage has been identified. This report brings our attention towards difficult differential diagnosis in emerging infections such as COVID-19.

## Clinical presentation

A 73-year-old male presented on March 25, 2020, to the emergency room for a 1-week history of mild influenza like symptoms which rapidly progressed to respiratory distress and high fever (38℃). He had no history of cardiovascular disease, hypertension or diabetes. Because of the constellation of findings in the context of ongoing acute pandemia, the patient was immediately admitted to our intensive care unit for suspected COVID-19).

## Investigations

**Figure 1.**
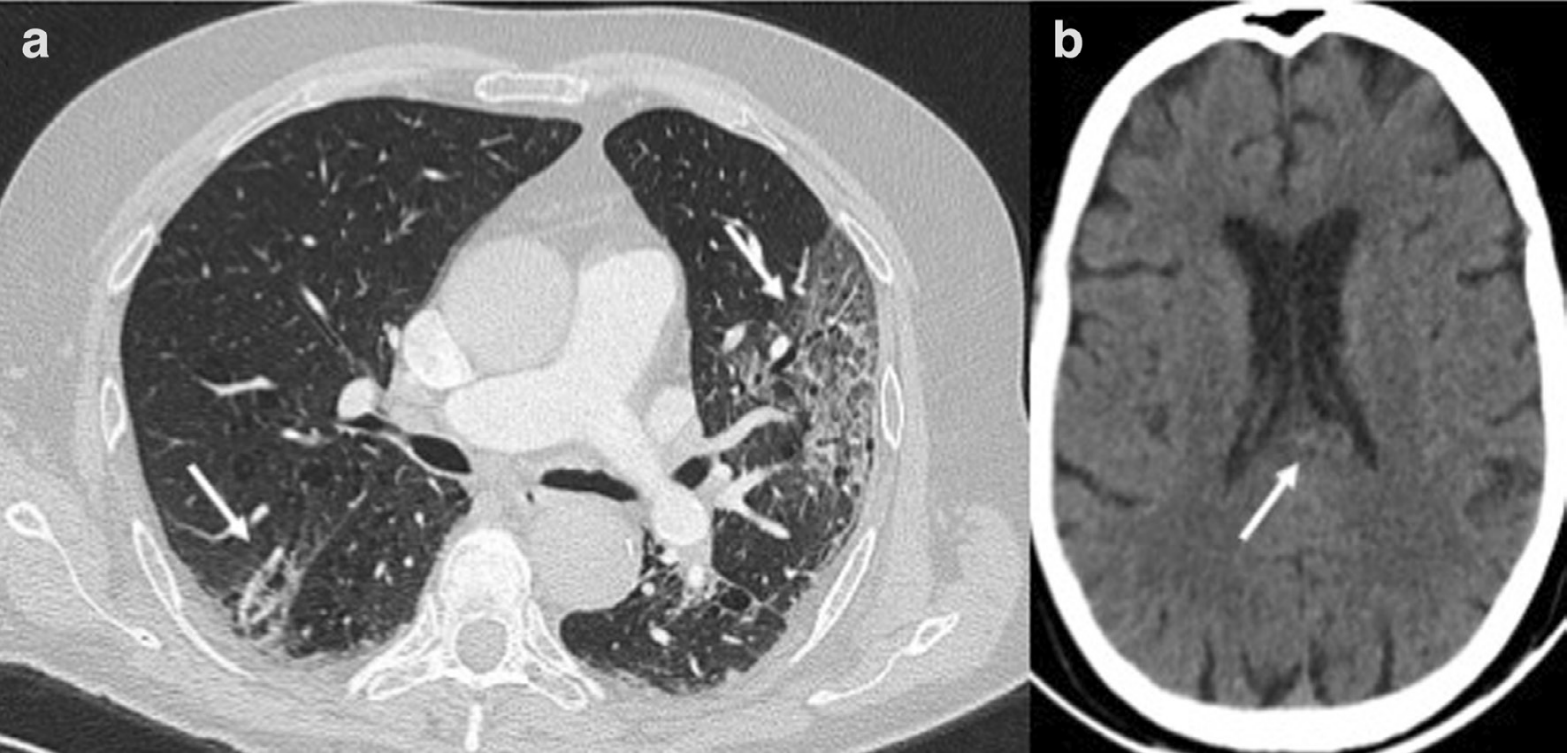
CT findings:( A) Chest CT shows bilateral ground glass opacities with a typical “crazy paving” pattern. (B) Brain CT shows a slightly hyperdense irregular lesion with slight mass effect on the medial wall of the occipital horn on the left lateral ventricle.

**Figure 2.**
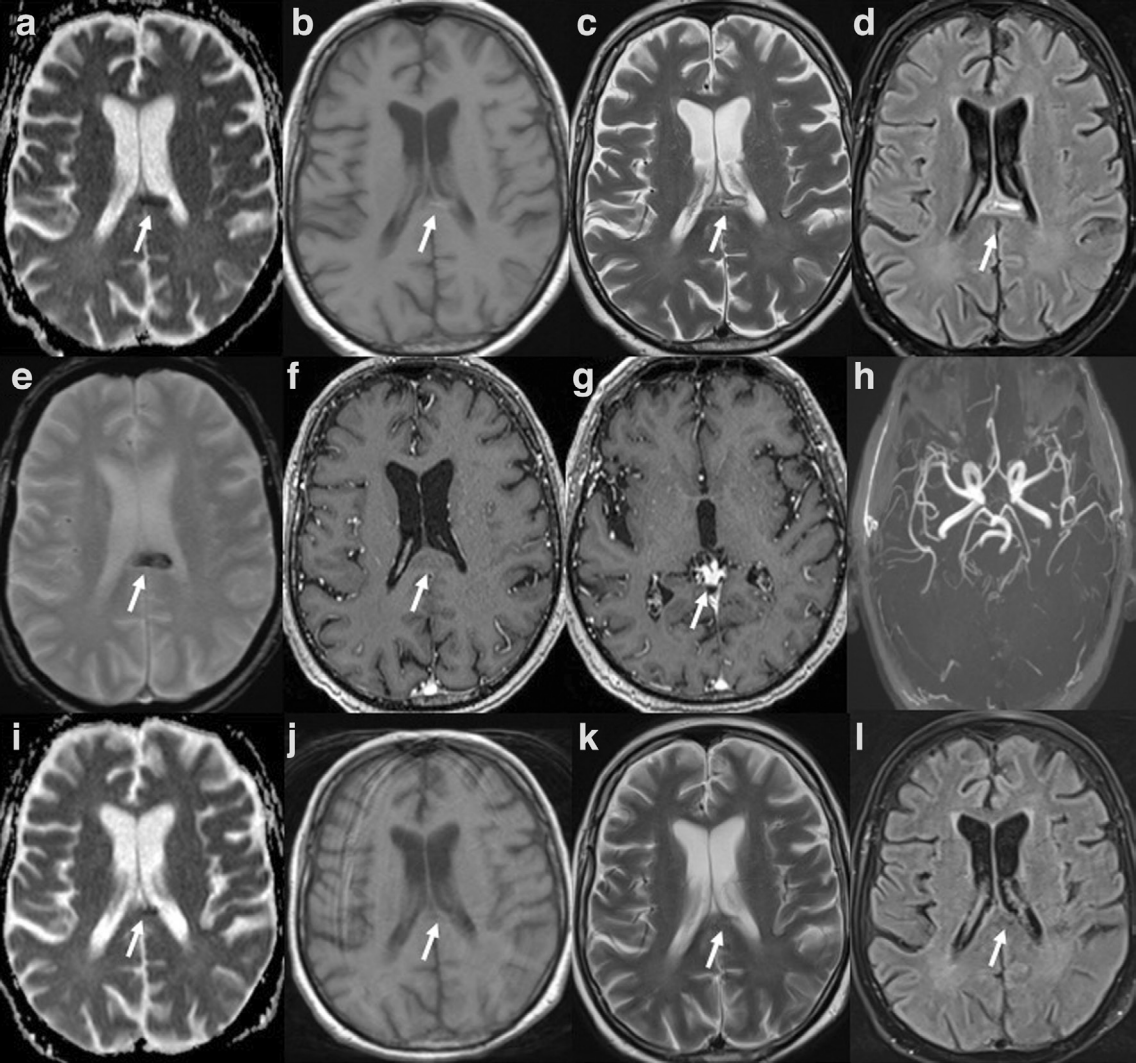
MRI findings in a 73-year-old male with COVID-19 (top two rows): (A) ADC) map shows a midline, hyporintense lesion in the splenium of the corpus callosum (CLOCC); (B) *T*_1_ weighted image showing slight increase in the signal; C) *T*_2_ weighted image show central hyperintensity with surrounding iso/hypointense rim; (D) in FLAIR, the lesion appears hyperintense, more hyperintense centrally; (E) the lesion in gradient-echo image appears hypointense; (F) no true gadolinium enhancement is noticed; (G) T1-MPRAGE images did not show any filling defect in the deep cerebral veins and (H) 3D-TOF was negative for cerebral aneurysms. MRI findings at follow-up (third row): (I) ADC map shows progressive residual area of restriction; (J) *T1*W image shows a small hypointense area; (K) *T2*W and (L) FLAIR show quasiresolution of the initial lesion likely evolving into a small malacic area. 3D-TOF, three-dimensional time of flight; ADC, apparent diffusion coefficient; FLAIR, fluid attenuated inversion recovery.

Initial laboratory exams revealed C-reactive protein levels of 20.1 mg/l (normal values:<6 mg/l) and a normal count of leukocytes and platelets. Nasopharyngeal swab specimen tested using the ﻿Real-Time Reverse Transcriptase-Polymerase Chain Reaction assay was positive for SARS-CoV-2 and COVID-19 was diagnosed. Both ferritin and D-dimer levels were elevated. Chest CT was positive for multiple bilateral pulmonary ground glass opacities with interstitial thickening (crazy-paving pattern) (Figure 1a). No signs of pulmonary embolism were found. After 3 weeks of intensive care therapy, with improved blood gas analysis, the patient continued to present altered consciousness. A brain CT was ordered which revealed a subtle, irregularly shaped hyperdense lesion within the splenium with mild mass effect on the medial wall of the lateral ventricle. This was suspected for hemorrhage (Figure 1b). Considered an unusual region for spontaneous hemorrhage, a brain MRI was performed the next day. An isolated lesion in the splenium slightly offset to the left, with a longitudinal morphology along the length of the splenial fibers was seen. MR characteristics of the lesion included: a hypointense lesion on apparent diffusion coefficient map (611.44 ± 90.66 mm^2^/s), slight hyperintensity on both *T1* weighted imaging, isohypointense on *T2* weighted imaging and hyperintense on fluid attenuated inversion recovery images with no gadolinium enhancement (Figure 2). The lesion on T2* image was hypointense. No other lesions were seen in the remaining supra and infratentorial structures. Three-dimensional-time of flight images were negative for aneurysm of the posterior circulation. To reduce the overall acquisition times, we did not perform a specific MR venogram. No filling defect was observed in the deep cerebral veins. The characteristics of our MR findings were presence of blood products in the subacute stage in the mid splenium. At this point, a cerebrospinal fluid (CSF) analysis was performed in order to exclude the presence of viral antigens or signs of inflammation such as an acute hemorrhagic encephalitis. No neurotropic viral antigens including those related to SARS-CoV-2 were found. CSF was clear and colorless. No cells were found, glucose was 3.6 mmol l^−1^ and protein was 0.38 g/l. No oligoclonal bands were found in the CSF or in the blood serum of the patient.

## Treatment

Severe hypoxia was treated with bronchial intubation and mechanical ventilation and sedation was achieved with intravenous benzodiazepines, propofol and opioids. The patient did not receive extracorporeal membrane oxygenation therapy. A combination of antiretroviral (Darunavir/Cobicistat), antibiotics and hydroxychloroquine were started. Anticoagulation prophylaxis was introduced with low-molecular weight enoxaparin. Additional supportive therapy such as adequate antipyretic drugs, hydration and intraparenteral nutrition were also provided.

## Differential diagnosis

Acute respiratory distress syndrome characterized by dry cough, high fever and respiratory distress can be caused by a variety of infectious agents, including influenza viruses (especially during the winter months typically between December and May). Acute respiratory distress syndrome can also be triggered by non-infectious diseases such as cardiovascular diseases, lung trauma and aspiration pulmonitis to a name a few.^3^ In fact, an accurate history, a simple blood test and a chest X-ray can easily narrow down the etiology. The ongoing pandemic phase and the presence of SARS-CoV-2 viral antigen in the nasal swab were found to be sufficient to make the diagnosis of COVID-19.

Isolated solitary lesions in the splenium of the corpus callosum have diverse etiologies including metabolic disorders, tumors, trauma, demyelinating diseases and stroke. Brain MRI is necessary to reach an appropriate diagnosis.^4^ However, because of increased risk of infection and unstable clinical conditions in the COVID-19 environment, brain imaging may be delayed.

Isolated splenial lesions that appear hyperintense on diffusion weighted images (DWI) are well-recognized and are an expression of excitotoxic damage to vulnerable splenial nerve fibres due to release of glutamate that is triggered by a variety of endogenous and exogenous *noxa*.^5^ Such lesions, also known as cytotoxic lesion in the splenium of the corpus callosum (CLOCC), are often in the midline and are generally reversible.^6,7^ CLOCCs have been found in association with influenza virus infections, several metabolic disorders, trauma, variety of pharmaceutical drug toxicity and metabolic disorders.^7^ From a neurological point of view, splenial lesions are characterized by altered consciousness and seizures that correlated well with our patient’s symptoms.^8^

These lesions are readily recognized on MR images as hyperintense on DWI and are generally non-enhancing in the acute phase. This together with the clinical history of the patient is often sufficient to make the diagnosis. However, a DWI hyperintense lesion can also represent a subacute hematoma or a hemorrhagic infarct.^9^ COVID-19 positive patients are known to be at high risk of thromboembolic events and stroke has been reported in such patients.^10^ However, a stroke in the splenium is a rather rare entity. Gradient recall echo *T2* weighted imaging did not show any other microhemorrhage or superficial cortical siderosis thereby excluding possible amyloidosis-related hemorrhage. Our patient also had very high levels of ferritin which is considered a proinflammatory cytokine; this may have initiated endothelial damage to vessels in the splenium. We interpreted our findings as a CLOCC that later hemorrhaged due to persistent presence of *noxa* damaging the blood–brain barrier in an anticoagulated patient, but a splenial artery hemorrhagic stroke cannot be dismissed. Prolonged use of multiple drugs such as propofol with antiepileptic properties could also have contributed to such a lesion.

## Outcome and follow-up

After 4 weeks of intensive care therapy, the patient was transferred to a normal ward with continuous monitoring of all vital parameters. Neuroimaging driven patient management included dosage reduction of sedatives, removal of mechanical ventilation and switching to non-invasive ventilation. No episodes of profound hypotension were recorded to warrant use of vasopressors. At 5 week follow-up, the patient was successfully weaned-off of ventilation. Follow-up MR showed the presence of focal residual hemosiderin deposits and myelomalacia in the former region of the hemorrhage (Figure 2).

## Discussion

This case provides several reflections for the radiologist/neuroradiologist who may find themselves frontline in the differential diagnosis of brain lesions in patients with COVID-19 on both CT and MRI. Firstly, SARS CoV-2 may enter the CNS, by directly invading the olfactory, trigeminal and the vagus nerves causing symptoms like hyposmia, dysgeusia and perhaps central respiratory depression.^11^ Experimental work using mice inoculated intranasally with the 2003 SARS-CoV demonstrated that viral antigens were present in the nucleus ambiguus and in the nucleus of the solitary tract in the medulla of mice but also in the thalamus.^12^ The authors proposed that the virus invaded the brain via a axonal/synaptin route reaching areas directly connected to the olfactory bulbs such as the hypothalamus, thalamus and the amygdala, a pattern of brain parenchymal hemorrhagic lesions reported recently in a patient with COVID-19.^13^ Secondly, SARS-CoV, may also invade the brain via angiotensin-converting enzyme 2 receptors that are located principally in the vascular endothelial cells and smooth muscle cells.^7^ As SARS CoV-2 enters lung host cells via the same receptor on the epithelial cells, it is likely that it uses a similar route of entry to the brain but this has not yet been established.^14,15^ Thirdly, a massive release of inflammatory cytokines, can cause brain inflammation similar to secondary ﻿hemophagocytic lymphohistiocytosis or acute disseminated encephalomyelitis or acute hemorrhagic encephalitis.^16,17^ More recently, it has been noted that SARS-CoV-2 can cause vasculitis and thromboembolic complications are frequent.^18^ And last but not the least, the use of multiple drugs in the acute phase, the possible development of vitamin deficiencies, malnourishment, dehydration, high fever, metabolic alterations, prolonged immobility and cerebral hypoperfusion in the severely acute COVID-19 patients, may all contribute to a variety of peripheral nervous system, brain and spine alterations often also seen in other toxic and metabolic diseases.^19,20^

Brain imaging in COVID-19 is still at its earliest stages at the time of writing. A constellation of non-specific MRI findings have been reported in COVID-19 positive patients such as stroke, leptomeningeal enhancement and posterior reversible encephalopathy.^10,21^ Also a case of acute necrotizing encephalopathy in a patient with COVID-19 has been reported so far, although it is not specific for COVID-19.^13,18^ Specificity of MR findings will require a large number of cases from different centers across the world in patients and perhaps with RT-PCR positive for SARS-CoV-2 in the CSF.

The major recognized limitation of this report is that it is a single case. While we want to stay away from reporting every single brain MRI finding in the novel COVID-19 outbreak, the attempt here is to recognize the several mechanisms that might contribute to brain MRI findings in COVID-19 patients and offer medical health professionals in every part of the world a point of reference in interpreting CT and MRI findings in COVID-19 positive patients.

## Learning points

1. COVID-19 is an emerging infection that is being rapidly recognized to affect several organs including the human brain.
2. Similar to influenza viruses, isolated lesions in the splenium is likely to be caused by “cytokine storm” triggered by SARS-CoV-2 and concomitant aggressive supportive therapy, although a wide differential diagnosis must be considered.
3. Neuroimaging must be considered in acute phases of the illness to detect brain lesions that can help guide therapy such as the use of anticoagulants.
